# Author Correction: Berberine is an insulin secretagogue targeting the KCNH6 potassium channel

**DOI:** 10.1038/s41467-021-26635-8

**Published:** 2021-10-29

**Authors:** Miao-Miao Zhao, Jing Lu, Sen Li, Hao Wang, Xi Cao, Qi Li, Ting-Ting Shi, Kohichi Matsunaga, Chen Chen, Haixia Huang, Tetsuro Izumi, Jin-Kui Yang

**Affiliations:** 1 Department of Endocrinology, Beijing Tongren Hospital, Capital Medical University, 100730 Beijing, China; 2 Beijing Key Laboratory of Diabetes Research and Care, Beijing Diabetes Institute, 100730 Beijing, China; 3 Laboratory of Molecular Endocrinology and Metabolism, Department of Molecular Medicine, Institute for Molecular and Cellular Regulation, Gunma University, Maebashi, Japan; 4 School of Biomedical Sciences, University of Queensland, Brisbane, QLD 4072 Australia; 5 Department of Physiology and Pathophysiology, School of Basic Medical Sciences, Capital Medical University, 100069 Beijing, China

**Keywords:** Ion transport, Diabetes, Diabetes, Drug development

Correction to: *Nature Communications* 10.1038/s41467-021-25952-2, published online 23 September 2021.

In this article the author name Tetsuro Izumi was incorrectly written as Testuro Izumi. The original article has been corrected.

