# Supplementary material for: Berberine is an insulin secretagogue targeting the KCNH6 potassium channel
Source: Nat Commun. 2021 Sep 23;12:5616. doi: 10.1038/s41467-021-25952-2 (PMC8460738; doi:10.1038/s41467-021-25952-2)
Supplement: Supplementary file 1 — Supplementary information [file 41467_2021_25952_MOESM1_ESM.pdf]

Supplementary information for

Berberine derived from a Chinese herb is a novel insulin secretagogue targeting the KCNH6 potassium channel

Miao-Miao Zhao[1,2], Jing Lu[1,2], Sen Li[2], Hao Wang[3], Xi Cao[1,2], Qi Li[2], Ting-Ting Shi[1,2], Kohichi Matsunaga[3], Chen Chen[4], Huang Haixia[5], Testuro Izumi[3] and Jin-Kui Yang[1,2]✉

[1]Department of Endocrinology, Beijing Tongren Hospital, Capital Medical University, Beijing 100730, China

[2]Beijing Key Laboratory of Diabetes Research and Care, Beijing Diabetes Institute, Beijing 100730, China

[3]Laboratory of Molecular Endocrinology and Metabolism, Department of Molecular Medicine, Institute for Molecular and Cellular Regulation, Gunma University, Maebashi, Japan

[4]School of Biomedical Sciences, University of Queensland, Brisbane 4072, Australia

[5]Department of Physiology and Pathophysiology, School of Basic Medical Sciences, Capital Medical University, Beijing 100069, China

✉Address correspondence and reprint requests to Professor Jin-Kui Yang, Department of Endocrinology, Beijing Tongren Hospital, Capital Medical University, Beijing 100730, China

E-mail: jkyang@ccmu.edu.cn

Supplementary information includes 12 figures, 4 tables and 2 notes.

Supplementary Figures

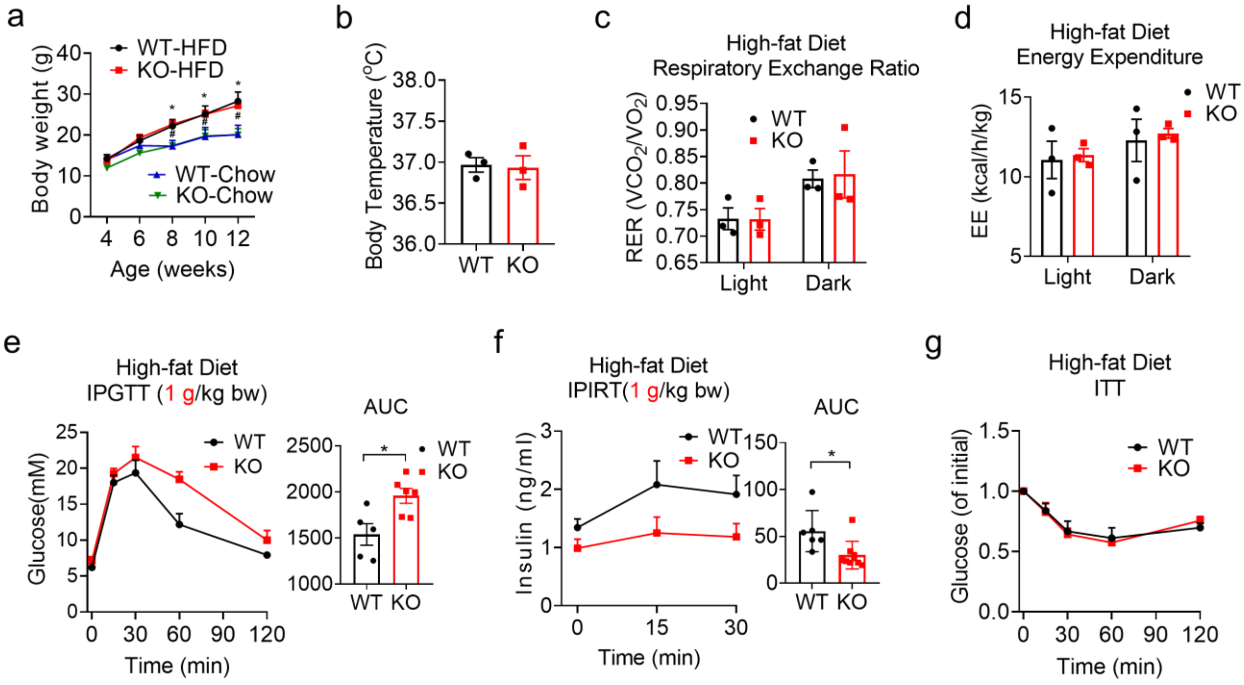

Supplementary Fig. 1: High-fat diet (HFD)-fed *Kcnh6* knockout (KO) mice exhibit impaired glucose tolerance.

a, Body weights of wild type (WT) and KO mice fed the HFD or chow diet from 4-12 weeks (n=8).8w: P = 0.043 for WT-HFD vs. WT-Chow, P = 0.048 for KO-HFD vs. KO-Chow; 10w: P = 0.029 for WT-HFD vs. WT-Chow, P = 0.028 for KO-HFD vs. KO-Chow; 12w: P = 0.026 for WT-HFD vs. WT-Chow, P = 0.027 for KO-HFD vs. KO-Chow. Statistical significance was assessed using one-way ANOVA with Tukey's *post hoc* test (two-sided).

b-d, Metabolic phenotypes of HFD-fed WT and KO mice at the age of 12 weeks. **(b)** Body temperature, **(c)** respiratory exchange rate and **(d)** energy expenditure of HFD-fed WT and KO mice.

e-f, HFD-fed WT and KO mice were loaded with 1 g/kg glucose at week 12. **(e)** Blood glucose (during intraperitoneal glucose tolerance test (GTT), n=5, P = 0.017) and **(f)** plasma insulin (during insulin release test (IRT), n=6, P = 0.018) levels were measured at the indicated times and were calculated as the area under the curve (AUC).

g, Blood glucose levels measured during the insulin tolerance test (ITT). The data are presented as the percentage of the glucose level at time 0 (0.75 IU insulin/kg bw; n=5).

The values are reported as means ± s.e.m. *P < 0.05. Statistical significance was assessed using the Mann-Whitney *U*-test (two-sided).

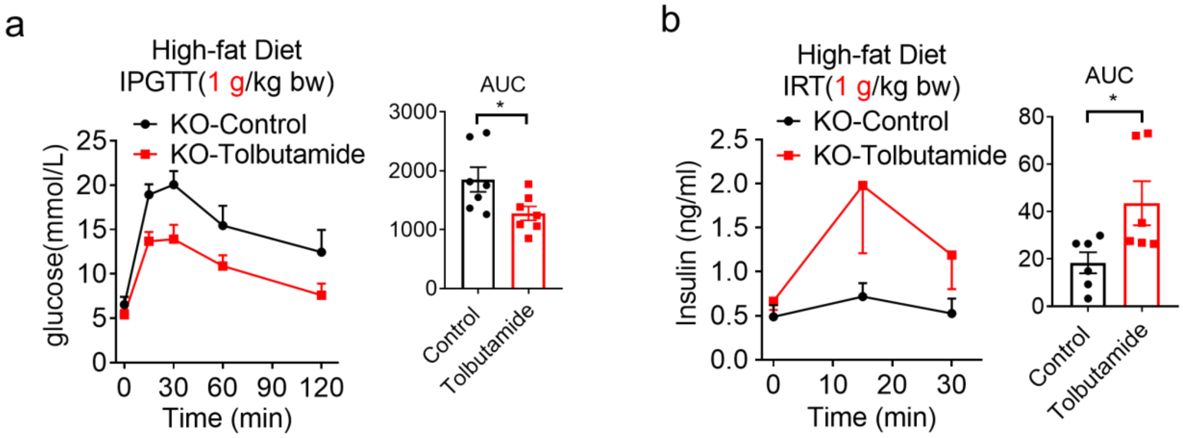

Supplementary Fig. 2: Tolbutamide increases insulin secretion in *Kcnh6* KO mice. HFD-fed KO mice were orally administered 40 mg/kg tolbutamide before being loaded with 1 g/kg glucose. **(a)** Blood glucose (during the intraperitoneal glucose tolerance test (GTT), n=7, P = 0.038) and **(b)** plasma insulin (during the insulin release test (IRT), n=6, P = 0.041) levels were measured at the indicated times and were calculated as the AUC. The values are presented as means ± s.e.m. *$P < 0.05$. Statistical significance was assessed using the Mann-Whitney U-test (two-sided).

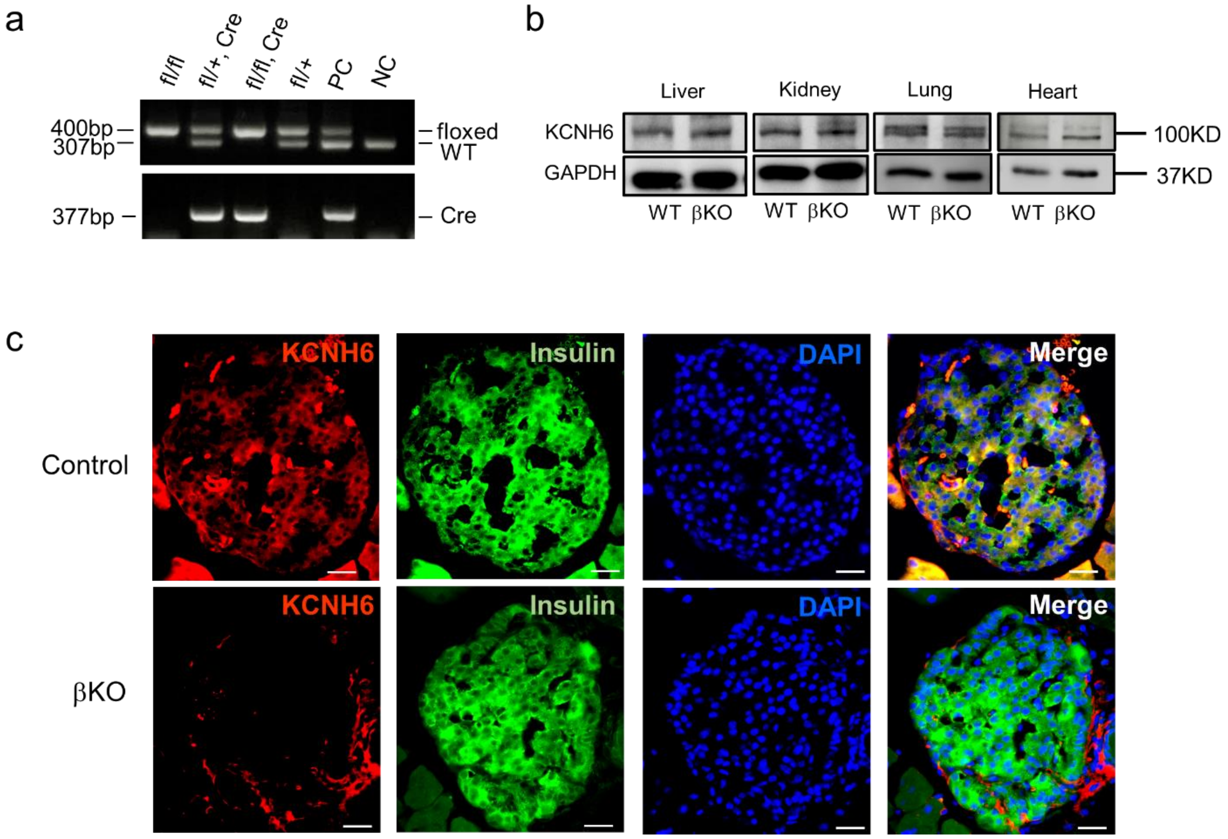

Supplementary Fig. 3: Tissue-specific ablation of *Kcnh6* in pancreatic islet β-cells.

a, PCR analysis of genomic DNA from tail clippings. The WT (307 bp), floxed (400 bp), and Cre (377 bp) PCR bands are indicated. The genotypes of representative littermates are indicated. fl, *Kcnh6*-floxed. PC, positive control. NC, negative control. A representative PCR analysis from 3 different experiments is shown.

b, Western blot analysis of levels of the KCNH6 protein in islets (Figure 2h), livers, kidneys, lungs and hearts of the control and the βKO mice using antibodies against KCNH6 and GAPDH. A representative immunoblot from 3 different experiments is shown.

c, Immunostaining of pancreatic sections from control and βKO mice with antibodies against KCNH6 and insulin (bar, 20 μm). Representative images of immunostaining from 3 different experiments are shown.

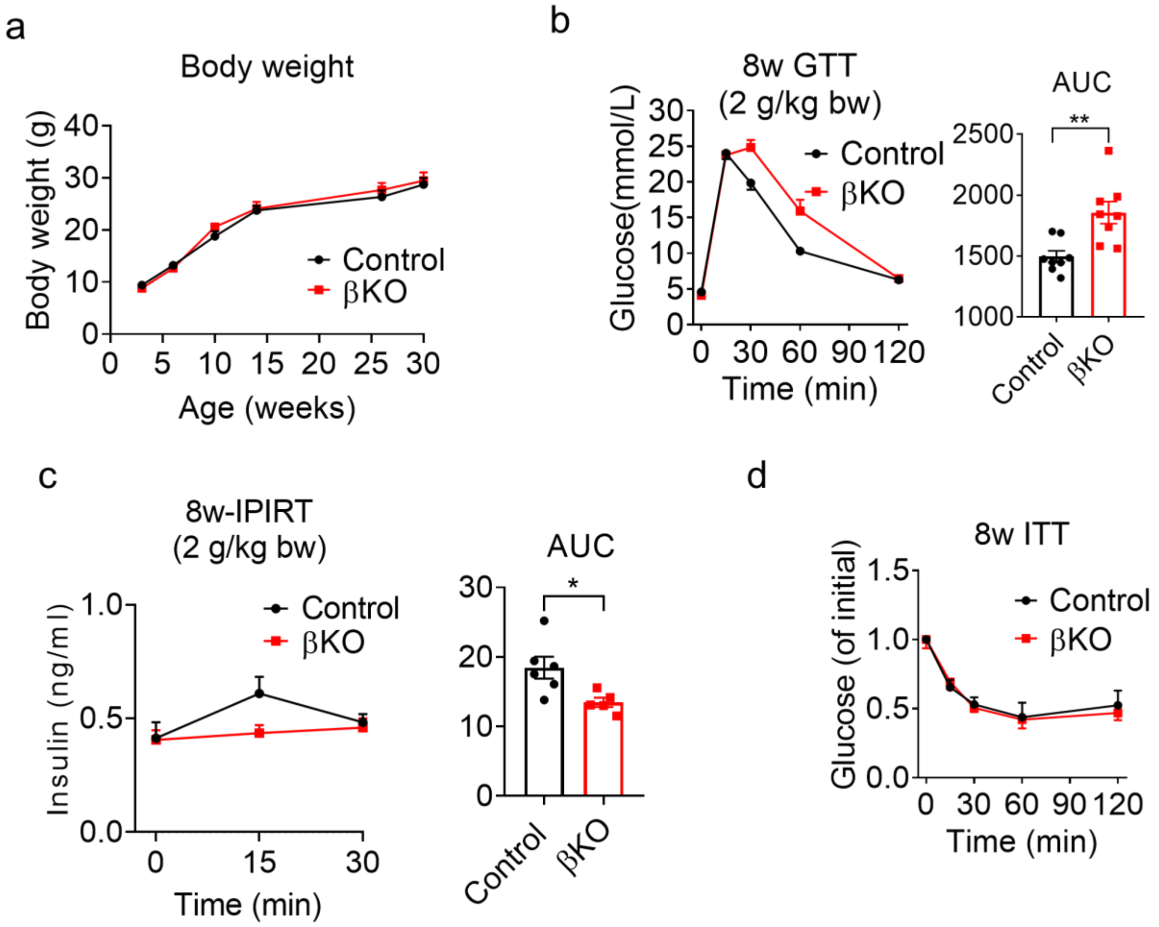

Supplementary Fig. 4: CRISPR-mediated βKO mice exhibit impaired glucose tolerance.

a, Body weights of control and βKO mice (n=12 for control and n=8 for βKO mice).

b, Blood glucose levels and AUCs at week 8, as determined using IPGTTs (2 g glucose/kg bw; n=8). $P = 0.002$.

c, Plasma insulin levels and AUCs at week 8, as determined using IPIRTs (2 g glucose/kg bw; n=6) $P = 0.024$.

d, Blood glucose levels at week 8, as determined using ITTs. The data are reported as the percentage of the glucose level at time 0 (0.75 IU insulin/kg bw; n=4).

The values are presented as means ± s.e.m. $*P < 0.05$ and $**P < 0.01$. Statistical significance was assessed using the Mann-Whitney *U*-test (two-sided).

a

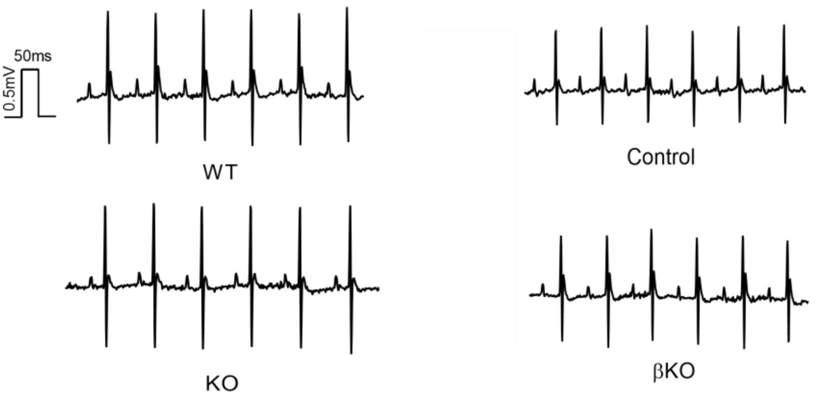

b

	WT (n=8)	KO (n=8)	*P*	Control (n=6)	βKO (n=6)	*P*
HR (bpm)	437.3 ± 22.1	421.2 ± 16.3	0.568	451.9 ± 14.6	431.0 ± 27.8	0.557
P duration (ms)	12.6 ± 0.8	12.3 ± 0.8	0.767	11.6 ± 0.8	10.4 ± 0.5	0.258
QRS (ms)	11.5 ± 1.2	10.3 ± 1.4	0.530	10.2 ± 0.3	10.1 ± 0.3	0.856
QTc (ms)	67.0 ± 3.7	68.8 ± 3.2	0.732	54.4 ± 1.2	53.5 ± 1.1	0.623

Data are representative as mean ± SEM.

Supplementary Fig. 5: The electrocardiogram (ECG) analysis of global KO and βKO mice

a, Representative ECG recorded from WT and global KO mice, and from control and βKO mice.

b, ECG measurements of the WT and global KO mice, and the control and βKO mice. HR, heart rate; QRS, QRS interval; QTc, corrected QT interval. Statistical significance was assessed using Student's *t* test (two-sided).

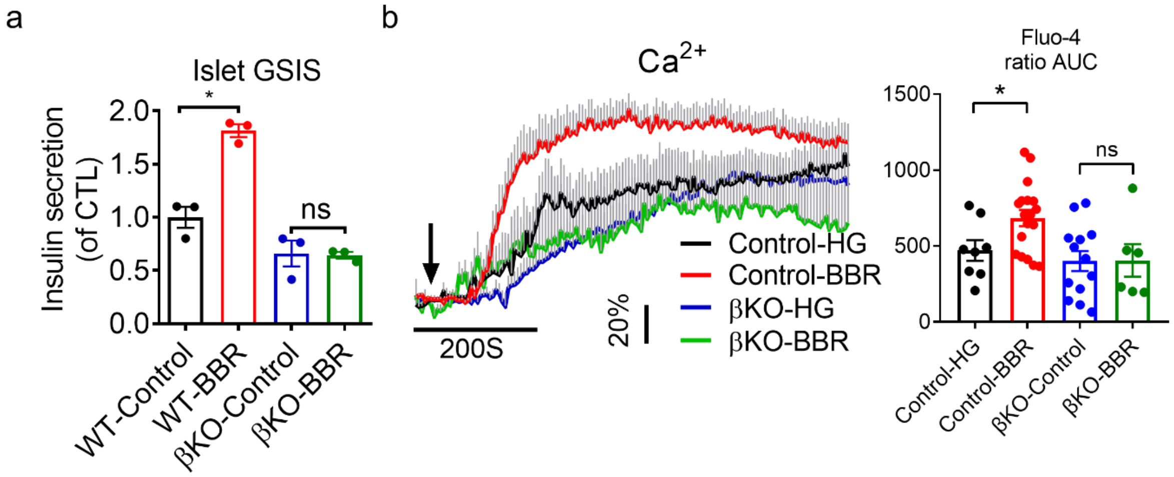

Supplementary Fig. 6: Effects of BBR on βKO islet β-cells *in vitro*.

a, Pancreatic islets from WT and βKO mice were treated with 5 μM BBR or the vehicle (control) while being stimulated with 25 mM glucose for 30 min. The secreted insulin level in the supernatant was normalized to the total insulin content in the islets. n=3. P = 0.022 for WT-Control vs. WT-BBR. Statistical significance was assessed using the Mann-Whitney U-test (two-sided).

b, Glucose stimulated an increase in the intracellular Ca^{2+} concentration in primary islet β-cells from control and βKO mice stimulated with 25 mM glucose and treated with or without 5 μM BBR as indicated (left panel), the calculated AUCs are shown (right panel). Intracellular Ca^{2+} levels were measured as the density of fluorescence and are reported as the percentage of basal levels (control-HG, n=8; control-BBR, n=18; βKO-HG, n=13; βKO-BBR, n=6). P = 0.047 for Control-HG vs. Control-BBR. Statistical significance was assessed using Brown-Forsythe and Welch ANOVAs with a Games-Howell *post hoc* test for multiple comparisons (two-sided).

The values are presented as means ± s.e.m. *P < 0.05, **P < 0.01, and ****P<0.0001.

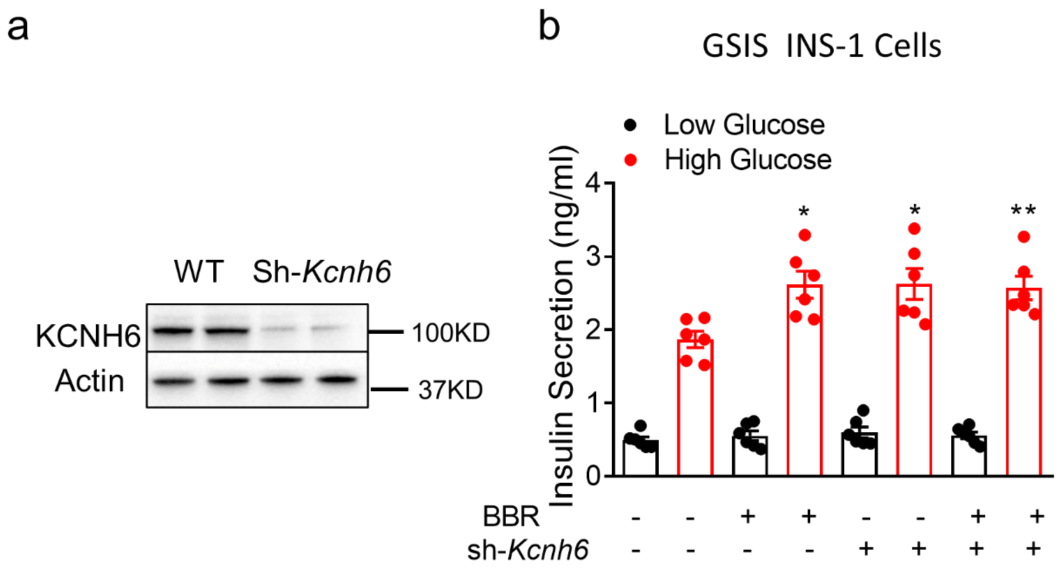

Supplementary Fig. 7: Effects of BBR on glucose-stimulated insulin section (GSIS) in INS-1 cells.

INS-1 cells were transfected with the *Kcnh6* shRNA (KD INS-1 cells) or scrambled shRNA (WT INS-1 cells).

a, Western blot analysis of levels of the KCNH6 protein in WT and KD INS-1 cells. A representative immunoblot from 3 different experiments is shown.

b, WT and KD INS-1 cells were treated with or without 10 μM BBR while being stimulated with 16.7 mM glucose for 30 min. The data are representative of six independent experiments. $P = 0.01$ for High glucose-BBR vs. High glucose; $P = 0.01$ for High glucose-sh-*Kcnh6* vs. High glucose; $P = 0.002$ for High glucose-BBR-sh-*Kcnh6* vs. High glucose. The values are presented as means ± s.e.m. $^*P < 0.05$. Statistical significance was assessed using the Mann-Whitney *U*-test (two-sided).

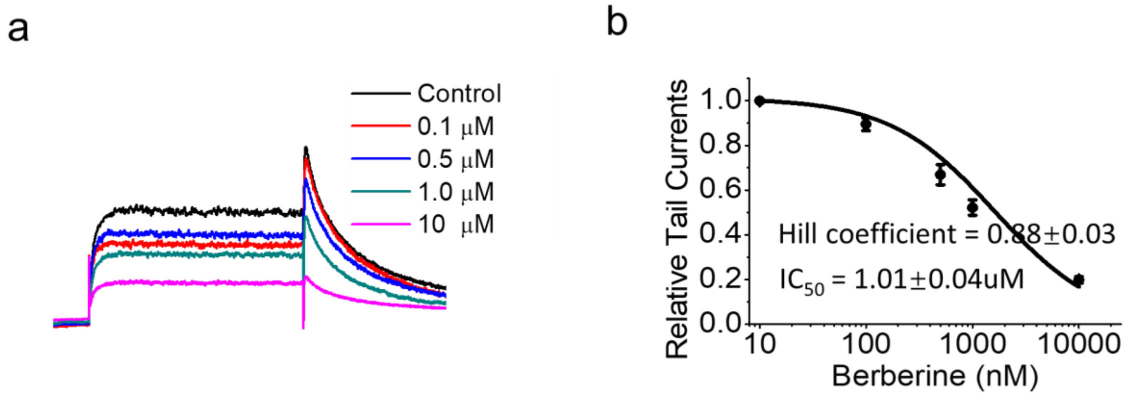

Supplementary Fig. 8: Dose-dependent effects of BBR on KCNH6 channel currents.

a, Representative currents in transfected HEK293T cells treated with the indicated concentrations of BBR.

b, Dose-dependent effects of BBR on the blockade of KCNH6 channels. Tail currents were determined from currents at +60 mM and are reported as a percentage of the responses recorded after 10 nM BBR application. IC_{50} = 1.01 ± 0.04 μM with a Hill coefficient of 0.88 ± 0.03. n=21 different cells.

The values are presented as means ± s.e.m.

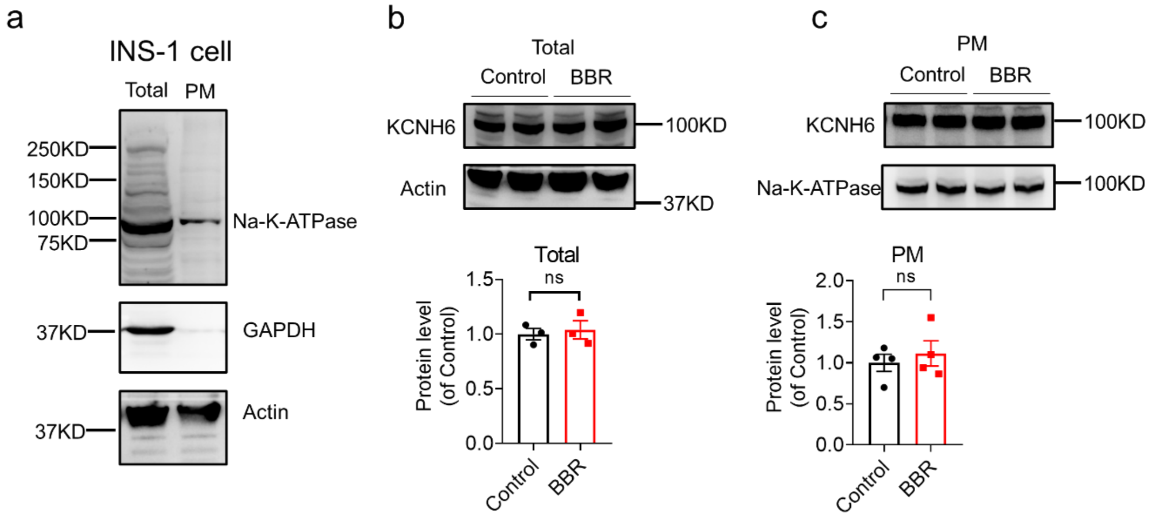

Supplementary Fig. 9: BBR inhibits KCNH6 channel currents by reducing the expression of the KCNH6 protein on the cell membrane.

a, The plasma membrane (PM) fraction and total cell lysates were isolated from INS-1 cells. Proteins were subjected to Western blotting using an antibody specific for Na-K-ATPase (PM marker), GAPDH (cytosol marker) and β-actin. A representative immunoblot from 3 different experiments is shown.

b, INS-1 cells were treated with the vehicle (control) or 10 μM BBR for 30 min as indicated. Proteins were subjected to Western blotting using an antibody specific for KCNH6. β-Actin was used as a loading control. A representative immunoblot from 3 different experiments is shown.

c, INS-1 cells were treated with the vehicle (control) or 10 μM BBR for 30 min as indicated. Proteins were subjected to Western blotting using an antibody specific for KCNH6. Na-K-ATPase was used as loading control. A representative immunoblot from 3 different experiments is shown.

The values are presented as means ± s.e.m. Statistical significance was assessed using the Mann-Whitney *U*-test (two-sided).

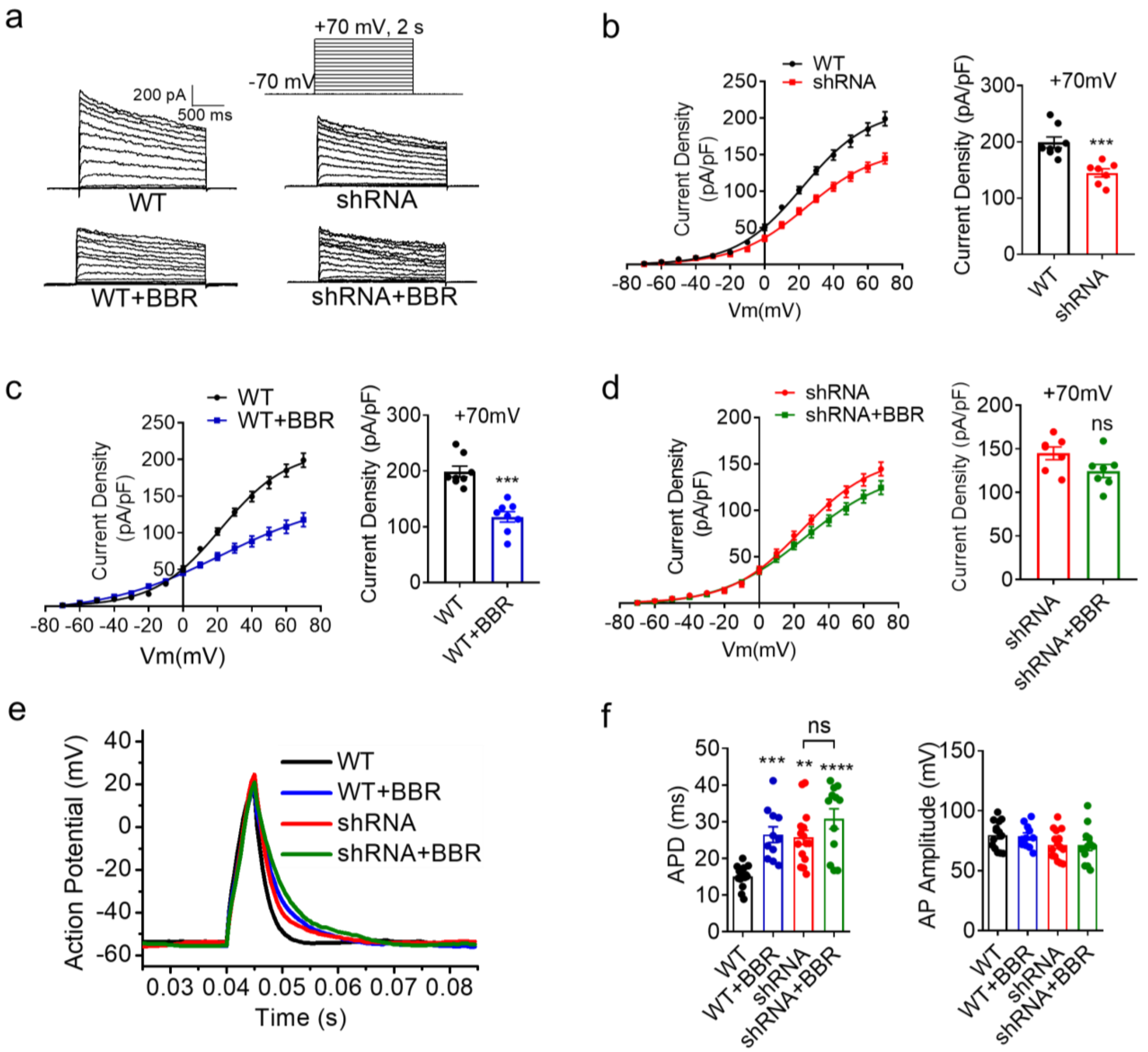

Supplementary Fig. 10: BBR inhibits K_v currents and prolongs action potential durations (APDs) in INS-1 cells.

a-d, K_v currents in 10 μM BBR- or vehicle-treated WT and KD (shRNA) INS-1 cells were recorded. **(a)** Representative K_v currents were recorded in WT and KD INS-1 cells treated as indicated, and **(b-d)** steady-state I-V curves for the K_v currents and mean the K_v current densities at +70 mV were calculated (WT, n = 8; KD, n=7). *P* = 0.0006 in **(b)**, *P* = 0.0002 in **(c)**. Statistical significance was assessed using the Mann-Whitney *U*-test (two-sided).

e-f, Action potentials of 10 μM BBR- or vehicle-treated WT and KD INS-1 cells. **(e)** Representative action potentials and **(f)** a summary of APDs and action potential amplitudes is shown (WT INS-1, n = 14; WT INS-1+BBR, n=11; KD INS-1, n=15; KD INS-1+BBR, n=12). *P* = 0.0009 for WT vs. WT+BBR, *P* = 0.001 for WT vs. shRNA, *P* <0.0001 for WT vs. shRNA+BBR in **(f)**. Statistical significance was assessed using one-way ANOVA with Tukey's *post hoc* test for multiple comparisons (two-sided).

The values are presented as means ± s.e.m. **P* < 0.05, ***P* < 0.01, ****P*<0.001, and *****P*<0.0001.

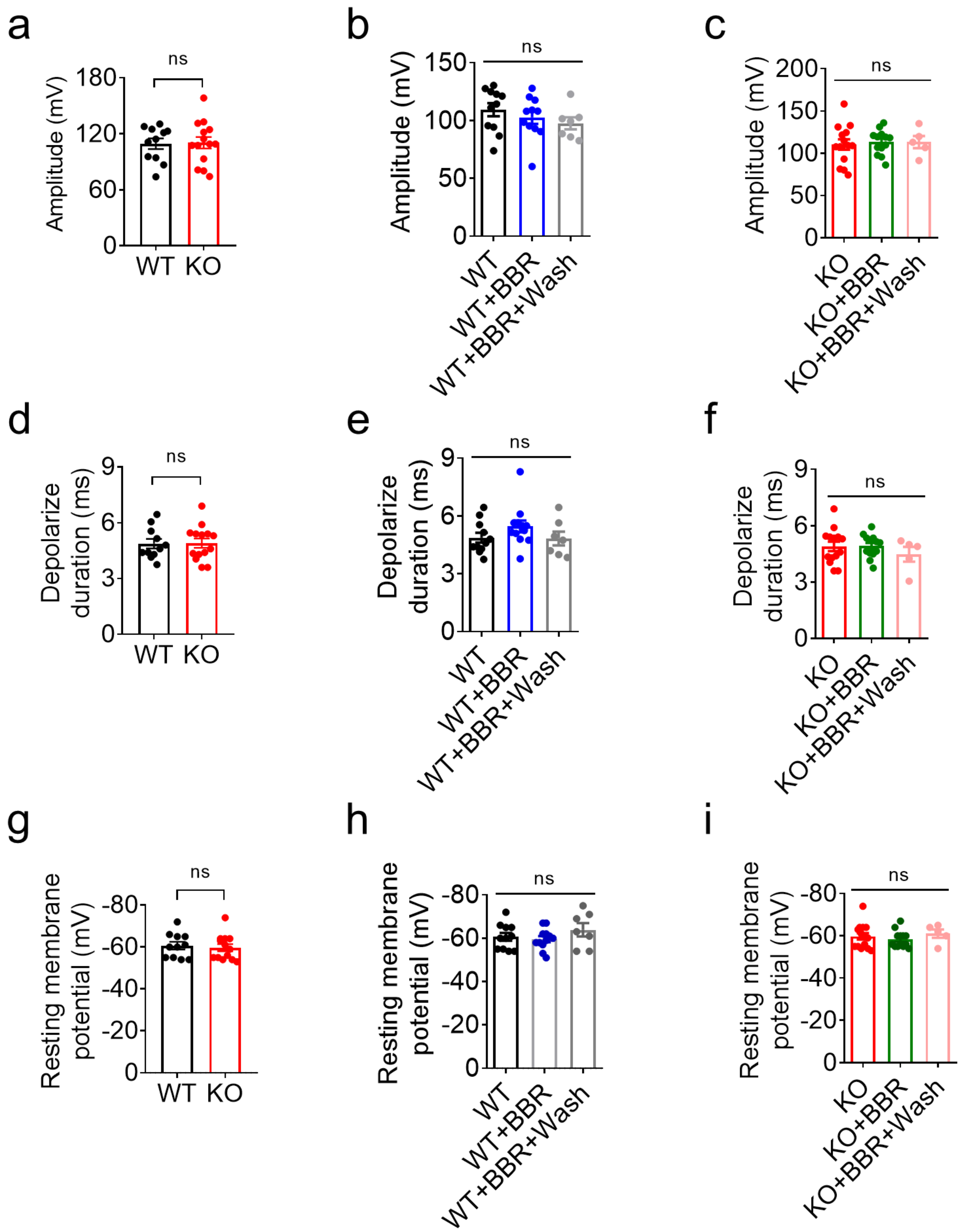

Supplementary Fig. 11: Effects of BBR on the electrophysiological characteristics of pancreatic islet β-cells from WT and KO mice.

a-c, Summary of action potential amplitudes in different cells.

d-f, Summary of depolarization durations in different cells.

g-i, Summary of resting potentials in different cells.

(WT, n=11; WT+BBR, n=11; WT+BBR+wash, n=7; KO, n=14; KO+BBR, n=13; KO+BBR+wash, n=5).

The values are presented as means ± s.e.m. Statistical significance was assessed using the Mann-Whitney *U*-test (two-sided) in a, d, and e and one-way ANOVA with Tukey's *post hoc* test for multiple comparisons (two-sided) for the remaining panels.

a

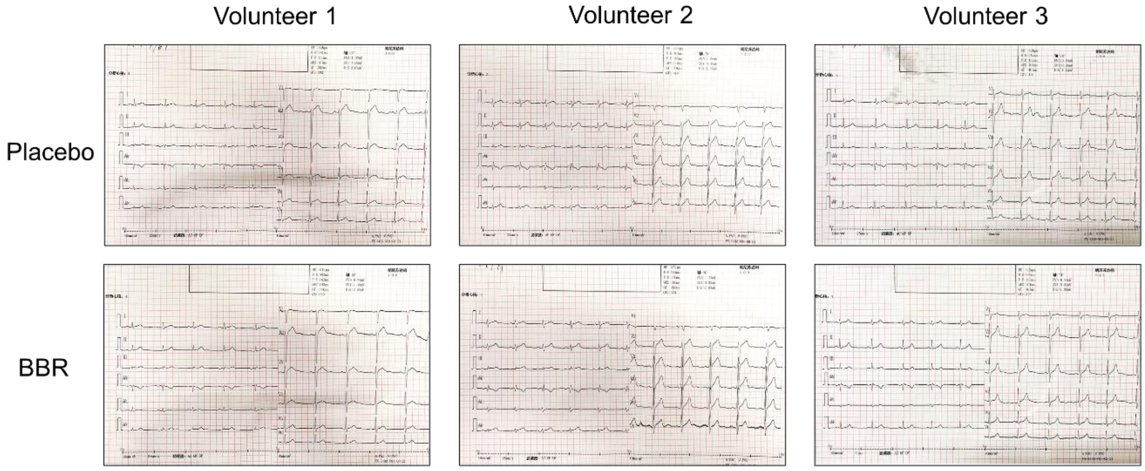

b

N = 15	Placebo	BBR	*P*
HR (bpm)	66.8 ± 1.6	67.6 ± 1.7	0.757
PR (ms)	144.3 ± 3.5	147.9 ± 3.9	0.504
QRS (ms)	107.4 ± 1.9	106.4 ± 2.1	0.721
QT (ms)	385.8 ± 5.5	387.6 ±5.6	0.820
QTc (ms)	406.0 ± 3.1	410.4 ± 3.5	0.374

Data are representative as mean ± SEM.

Supplementary Fig. 12: The ECG analysis from volunteers after the hyperglycemic study.

a, Representative ECG recorded from three volunteers receiving placebo or BBR. The volunteers were given placebo or BBR, and then performed the hyperglycemic clamp study, the ECG was performed after the hyperglycemic clamp. After the wash-out period, each volunteer received the other drug (BBR/placebo) and performed the ECG again.

b, ECG measurements of volunteers receiving placebo or BBR. HR, heart rate; PR, PR interval; QRS, QRS interval; QT, QT interval; QTc, corrected QT interval. Statistical significance was assessed using Student's *t* test (two-sided).

Supplementary Tables

Supplementary Table 1: Baseline characteristics of the 15 male subjects

	Mean	SD
Age (years)	30.63	4.83
Body mass index (kg/m^2)	21.37	1.30
Waist-to-hip ratio	0.83	0.03
Clinical measures		
Systolic blood pressure (mmHg)	118.50	7.83
Diastolic blood pressure (mmHg)	76.13	6.35
Laboratory results		
HbA1c (%)	5.36	0.33
Fasting blood glucose (mmol/L)	5.14	0.31
2-hr postprandial blood glucose (mmol/L)	5.07	0.55
Fasting plasma insulin (μIU/mL)	5.82	1.85
2-hr postprandial plasma insulin (μIU/mL)	34.57	21.82
Fasting plasma C-peptide (ng/mL)	1.55	0.34
2-hr postprandial plasma C-peptide (ng/mL)	6.15	2.35
Total cholesterol (mmol/L)	4.16	0.72
HDL cholesterol (mmol/L)	1.39	0.18
LDL cholesterol (mmol/L)	2.42	0.63
Triglycerides (mmol/L)	0.81	0.36

Supplementary Table 2: Results from the hyperglycemic clamp study

	Placebo	BBR	*P*
Fasting blood glucose level-pretreatment (mmol/L)	4.87 ± 0.35	4.96 ± 0.27	NS
Fasting blood glucose level-posttreatment (mmol/L)	5.05 ± 0.22	5.11 ± 0.31	NS
Fasting plasma C-peptide level-posttreatment (ng/mL)	1.48 ± 0.29	1.65 ± 0.43	NS
Fasting plasma insulin level-posttreatment (μIU/mL)	5.39 ± 1.61	6.39 ± 2.11	NS
Steady-state blood glucose level (mmol/L)	11.92 ± 0.27	12.01 ± 0.29	NS
Coefficient of variation (%)	4.87 ± 1.34	5.57 ± 2.17	NS
GIR (mg/kg/min)	16.98 ± 2.21	17.54 ± 3.55	NS

Pretreatment and posttreatment fasting blood glucose levels indicate the blood glucose levels of volunteers before/after taking the placebo/BBR while not receiving the glucose injection. Data are presented as means ± SD.

Supplementary Table 3: Antibodies used in this paper

Antibody	Company	Cat. No.	RRID
FLAG	Sigma-Aldrich, St. Louis, MO, USA	F3165	AB_259529
β-actin	Cell Signaling Technology, Danvers, MA, USA	58169	AB_42750839
GAPDH	Sigma-Aldrich, St. Louis, MO, USA	G9545	AB_796208
KCNH6	Sigma-Aldrich, St. Louis, MO, USA	SAB2104242	SAB2104242
Insulin	Abcam, Burlingame, CA, USA	ab6995	AB_305690
Na-K-ATPase	Cell Signaling Technology, MA, USA	3010S	AB_2060983
Alexa Fluor 555 goat anti-rabbit IgG	Invitrogen, South San Francisco, CA, USA	A32732	AB_2633281
Alexa Fluor 488 goat anti-mouse IgG	Invitrogen, South San Francisco, CA, USA	A32723	AB_2633275

Supplementary Table 4: Primers used to identify the *Kcnh6* βKO mice.

Primer	Forward	Reverse
Kcnh6	CCCCATCTGCTAAGCCTTAATTAC	GAAGCTGGAGGCTGCAAACC
Cre	GCCTGCATTACCGGTCGATGC	CAGGGTGTTATAAGCAATCCC

Supplementary Notes

Supplementary Note 1: CONSORT checklist

CONSORT 2010 checklist of information to include when reporting a randomised trial*

Section/Topic	Item No	Checklist item	Reported on page No
**Title and abstract**			
	1a	Identification as a randomised trial in the title	12
	1b	Structured summary of trial design, methods, results, and conclusions (for specific guidance see CONSORT for abstracts)	12
**Introduction**			
Background and objectives	2a	Scientific background and explanation of rationale	11-12
	2b	Specific objectives or hypotheses	11-12
**Methods**			
Trial design	3a	Description of trial design (such as parallel, factorial) including allocation ratio	12
	3b	Important changes to methods after trial commencement (such as eligibility criteria), with reasons	N/A
Participants	4a	Eligibility criteria for participants	24
	4b	Settings and locations where the data were collected	24
Interventions	5	The interventions for each group with sufficient details to allow replication, including how and when they were actually administered	24-25
Outcomes	6a	Completely defined pre-specified primary and secondary outcome measures, including how and when they were assessed	25-26
	6b	Any changes to trial outcomes after the trial commenced, with reasons	N/A
Sample size	7a	How sample size was determined	24
	7b	When applicable, explanation of any interim analyses and stopping guidelines	N/A
Randomisation:			
Sequence generation	8a	Method used to generate the random allocation sequence	25
	8b	Type of randomisation; details of any restriction (such as blocking and block size)	25
Allocation concealment	9	Mechanism used to implement the random allocation sequence (such as sequentially numbered containers), describing any steps taken to conceal the sequence until interventions were assigned	25

mechanism			
Implementation	10	Who generated the random allocation sequence, who enrolled participants, and who assigned participants to interventions	24-25
Blinding	11a	If done, who was blinded after assignment to interventions (for example, participants, care providers, those assessing outcomes) and how	24
	11b	If relevant, description of the similarity of interventions	N/A
Statistical methods	12a	Statistical methods used to compare groups for primary and secondary outcomes	26
	12b	Methods for additional analyses, such as subgroup analyses and adjusted analyses	N/A
**Results**			
Participant flow (a diagram is strongly recommended)	13a	For each group, the numbers of participants who were randomly assigned, received intended treatment, and were analysed for the primary outcome	25
	13b	For each group, losses and exclusions after randomisation, together with reasons	N/A
Recruitment	14a	Dates defining the periods of recruitment and follow-up	24
	14b	Why the trial ended or was stopped	N/A
Baseline data	15	A table showing baseline demographic and clinical characteristics for each group	Table S1.
Numbers analysed	16	For each group, number of participants (denominator) included in each analysis and whether the analysis was by original assigned groups	24
Outcomes and estimation	17a	For each primary and secondary outcome, results for each group, and the estimated effect size and its precision (such as 95% confidence interval)	11-13
	17b	For binary outcomes, presentation of both absolute and relative effect sizes is recommended	N/A
Ancillary analyses	18	Results of any other analyses performed, including subgroup analyses and adjusted analyses, distinguishing pre-specified from exploratory	N/A
Harms	19	All important harms or unintended effects in each group (for specific guidance see CONSORT for harms)	N/A
**Discussion**			
Limitations	20	Trial limitations, addressing sources of potential bias, imprecision, and, if relevant, multiplicity of analyses	14
Generalisability	21	Generalisability (external validity, applicability) of the trial findings	14
Interpretation	22	Interpretation consistent with results, balancing benefits and harms, and considering other relevant evidence	11-14
**Other information**			
Registration	23	Registration number and name of trial registry	11
Protocol	24	Where the full trial protocol can be accessed, if available	Supplementar

			y Note 2.
Funding	25	Sources of funding and other support (such as supply of drugs), role of funders	32-33

*We strongly recommend reading this statement in conjunction with the CONSORT 2010 Explanation and Elaboration for important clarifications on all the items. If relevant, we also recommend reading CONSORT extensions for cluster randomised trials, non-inferiority and equivalence trials, non-pharmacological treatments, herbal interventions, and pragmatic trials. Additional extensions are forthcoming: for those and for up to date references relevant to this checklist, see www.consort-statement.org.

Supplementary Note 2:

Research plan of the insulinotropic effect of berberine in human

1. Project purpose

By enrolling healthy subjects, they were treated with berberine (BBR) / placebo intervention, followed by the gold standard test for measuring insulin secretion, hyperglycemic clamp study, and glucose tolerance test to verify the effect of BBR in promoting insulin secretion.

2. Background information

BBR is a traditional Chinese medicine and the main active ingredient of the traditional Chinese herbal medicine *Coptis*. It has been used in Chinese medicine to treat diabetes for thousands of years. In recent years, many studies have shown that BBR has a significant effect in the treatment of diabetic patients. It can significantly reduce the blood glucose levels of diabetic patients, reduce HbA1c by 0.47-0.97%, and has multiple effects such as weight loss and blood lipid reduction without serious side effects. However, although BBR is safe and effective for the treatment of diabetes and is expected to be used as a new oral hypoglycemic agent in the clinic, its exact hypoglycemic mechanism is still unclear, which undoubtedly limits the clinical development of this excellent traditional Chinese medicine.

In recent years, our research group has devoted itself to studying the exact hypoglycemic mechanism of BBR, trying to unravel the mystery of this traditional Chinese medicine, and make the excellent Chinese herbal medicines speak out in the world. Our previous work found that BBR can promote pancreatic β-cell function and insulin secretion in mice *in vivo* and *in vitro*, thereby achieving the effect of lowering blood glucose.

3. Research progress and cases of hyperglycemic clamp study that can be used for reference

3.1 Current status of BBR used in clinical intervention trials

BBR has been used in clinical applications to treat diarrhea and gastrointestinal infections, but it also has good safety in patients with metabolic diseases such as diabetes and hyperlipidemia. As of 2019, BBR has been used in 37 clinical intervention trials for the treatment of diabetic patients. The following figure shows a meta-analysis of a clinical intervention trial of BBR published in 2015 (*1*). It shows that the application of BBR is safe in patients with diabetes, hyperlipidemia, and hypertension. According to related literature, except for individual gastrointestinal side effects, BBR has not reported other side effects. In addition, BBR is not only widely used in our country, but also widely used in the treatment of diabetes in India (*2*), which can at least prove the safety of the drug in clinical applications.

Table 1

Included trials	Sample size Q6	Testing scheme	Intervention measures (Test group/control group) (1/2/3)			Duration (days)	Outcomes	Jadad scores
Trials of berberine in the treatment of type 2 diabetes mellitus (17 trials)								
Yin et al. (2008)	15/16	RAN	BER, LI		METF, LI	91	ABCDEFGI	5
Gu et al. (2010)	30/30	CEN	BER		PLACEBO	90	ABCDEFGH	5
Zhang et al. (2008)	58/52	CEN	BER, LI		PLACEBO, LI	84	ABCDEFGHI	5
Cao et al. (2012)	38/40	RAN	BER, METF		METF	112	ABCDEFGI	3
Ding et al. (1996)	21/22	UNKN	BER		PHE	60	ABI	2
Liu and Hu (2008)	30/30	NUM	BER, MET, LI		METF, LI	56	ABC	4
Ning et al. (2013)	22/22	BLI	BER, METF, LI		METF, LI	112	ACDEI	4
Ren (2008)	31/30	RAN	BER, LI		LI	84	ABCDEFGI	3
Sheng and Xie (2010)	30/30	RAN	BER, GLIP, METF		GLIP, METF	90	AI	3
Ye (2009)	40/40	RAN	BER, GLIM, MET		GLIM, MET	90	ABCDEFGI	3
Zhang et al. (2011)	30/30	RAN	BER, LI		ROS, LI	90	ACDEF	3
Zhang et al. (2010)	50/26/21	RAN	BER	METF	ROS	60	ACDI	4
Cao (2007)	30/30/30	RAN	LI	METF, LI	BER, LI	90	ABCDEFGI	3
Jin (2014)	40/40/40	RAN	METF	BER	NAT	UNKN	DEFGHI	2
Li and Liu (2007)	50/51/51	RAN	GLIP	BER	GLIP, BER	60	ABCDEFGI	3
Xiang et al. (2011)	20/20/20	RAN	LI	ASP, LI	BER, LI	84	ABCDEFI	3
Zhu et al. (2008)	55/55/50	RAN	BER, LI	BER, MET, LI	METF, LI	90	ABCI	3
Trials of berberine in the treatment of hyperlipidemia (six trials)								
Su et al. (2012)	60/60	RAN	BER, SIM		SIM	56	DEFGI	2
Yu et al. (2007)	50/50	RAN	BER, SIM		SIM	84	DEFI	3
Zhou and Huang (2011)	60/60	RAN	BER, LI		LI	120	DEFG	3
He et al. (2007)	38/38/40	RAN	SIM	BER	SIM, BER	90	DEFGI	3
Wei et al. (2003)	34/16/18	UNKN	BER	SIM	ATO	60	DEFG	2
Zheng et al. (2009)	33/33/33	RAN	SIM	BER	BER, SIM	56	DEFGI	3
Q7 Trials of berberine in the treatment of hypertension (four trials)								
Huang (2013)	84/80	NUM	BER, AML		AML	56	DEFGH	4
Sun et al. (2013)	32/32	RAN	BER, AML		AML	56	DEFGH	3
Zhong et al. (1997)	96/96	UNKN	BER		NIT	28	HI	2
Han et al. (1999)	55/50/55	RAN	BER	METO	METO	28	HI	3

Note – BER: berberine; LI: lifestyle intervention; UNKN: unknown; METF: Metformin; ROS: rosiglitazone; SIM: simvastatin; GLIP: glipizide; AML: amlodipine; PHE: phenformin; NIT: nitrendipine; GLIM: glimepiride; NUM: the method of random number; CEN: center randomized double blind; BLI: randomized controlled double blind principle; ASP: aspilin; METO: metoprolol; SIM: simvastatin; ATO: atorvastatin; NAT: nateglinide; A: FPG; B: PPG; C: HbA_{1c}; D: TC; E: TG; F: LDL-C; G: HDL-C; H: BP; I: adverse reactions.

CORRECTED PROOF

3.2 Cases of hyperglycemic clamp study

After being described by Andres et al. in 1966, the hyperglycemic clamp study is considered to be the gold standard test for glucose homeostasis insulin measurement. It is widely used worldwide and has good safety. In 1997, Ligtengurg et al. (*3*) applied a hyperglycemic clamp study to detect the effect of the sulfonylurea drug, glibenclamide, on insulin secretion. The study enrolled 12 healthy young men who were given oral glibenclamide or placebo. After the administration, a hyperglycemic clamp study was performed to maintain the blood glucose level at 32 mmol/L for 2 hours. During the process, arterialized venous blood was drawn intermittently to measure blood glucose and insulin levels. In 2018, Shankar et al. (*4*) also selected 12 healthy male subjects to take sitagliptin or placebo orally and then perform a high-glucose clamp test to maintain blood glucose at 8.9 mmol/L for 6 hours, and draw venous blood for testing Insulin and C-peptide values, and cross-over design, each subject repeated the test 3 times, took 2 oral medications, 1 oral placebo test, wash-out interval of 1 weeks.

4. The main research content and technical route of the project

4.1 Main research content

4.1.1 This study screened healthy adult male subjects and the criteria for inclusion are:

1) Volunteer to participate in the trial and sign an informed consent form;

2) Healthy male subjects aged 18-45 (including 18 and 45 years old);

3) There is no current or previous history of diseases such as heart, liver, kidney, digestive tract, nervous system, respiratory system, mental disorders, and metabolic disorders that the investigator considers meaningful; no physical examination, electrocardiogram, and laboratory examination results abnormality or abnormality has no clinical significance (subject to the judgment of the physician);

4) The body mass index is 18.0-24.0kg/m^2 (including 18.0 and 24.0 kg/m^2);

5) Normal glucose tolerance (fasting blood glucose <6.1mmol/L, and 2h blood glucose <7.8mmol/L after oral administration of 75g glucose), insulin secretion function is normal (researchers judged by the results of insulin release experiment);
6) No family history of diabetes and obesity;
7) Able to communicate well with researchers and complete research in accordance with research regulations.
Exclusion criteria are:
1) Infection with hepatitis (A, B, or C), HIV and syphilis.
2) History of allergic reaction to berberine or any component in the formulation of the study drugs.
3) Cumulative amount of blood loss (eg. blood donation) over 400mL within 3 months prior to baseline visit and during the study.
4) Alcohol drinking within 2 weeks prior to baseline visit and during the study.
5) Use of illegal drugs or positive in urine drugs screen.
6) Smoke during the study.
4.1.2 This study is a randomized, 2-period, double-crossover test: the subjects are randomly divided into two groups with equal numbers of people. The order of administration of the first group is the first cycle of oral BBR, and the second cycle of oral placebo. The second group is administered with placebo in the first cycle and BBR in the second cycle; it will be eluted for at least 14 days between the two cycles.
4.2 Technical route of the project

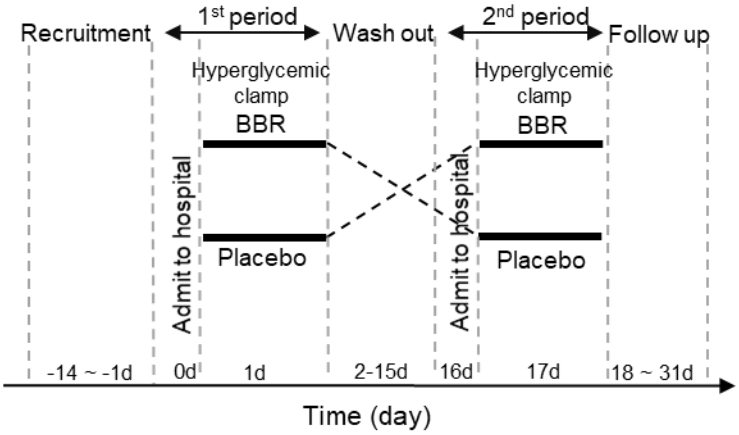

4.3 The main clinical indicators tested in this project
Inclusion criteria check, demographic data, medical history inquiry, exact screening, drug abuse screening, hepatitis B, hepatitis C, HIV, syphilis testing, coagulation routine, OGTT + IRT, glycosylated hemoglobin, blood routine, blood biochemistry, urine routine, ECG, Height, weight, waist circumference, bust circumference, physical examination, hyperglycemic clamp test
4.4 Outcome Measure:
Primary Outcome Measure:
1. Differences of serum insulin levels between BBR and placebo treatment groups during the hyperglycemic clamp study. To compare the mean serum insulin levels in the two groups during hyperglycemic clamp study.
2. Differences of serum C-peptide levels between BBR and placebo treatment groups during the

hyperglycemic clamp study. To compare the mean serum C-peptide levels in the two groups during hyperglycemic clamp study.

Secondary Outcome Measure:

3. Differences of glucose infusion rates between BBR and placebo treatment groups during the hyperglycemic clamp study. To compare the mean glucose infusion rates in the two groups during hyperglycemic clamp study.
4. Differences of blood glucose levels between BBR and placebo treatment groups during the hyperglycemic clamp study. To compare the mean blood glucose levels in the two groups during hyperglycemic clamp study.

Other pre-specified Outcome Measures:

5. Heart rate and QT-interval duration using electrocardiogram after drug treatment.

References:

1. J. Lan, Y. Zhao, F. Dong, Z. Yan, W. Zheng, J. Fan, G. Sun, Meta-analysis of the effect and safety of berberine in the treatment of type 2 diabetes mellitus, hyperlipemia and hypertension. *Journal of ethnopharmacology* **161**, 69-81 (2015); published online EpubFeb 23 (10.1016/j.jep.2014.09.049).
2. A. Luthra, A. Misra, The marketing of unproven drugs for diabetes and dyslipidaemia in India. *The Lancet Diabetes & Endocrinology* **3**, 758-760 (2015)10.1016/s2213-8587(15)00328-9).
3. J. J. Ligtenberg, C. E. Venker, W. J. Sluiter, W. D. Reitsma, T. W. Van Haeften, Effect of glibenclamide on insulin release at moderate and high blood glucose levels in normal man. *European journal of clinical investigation* **27**, 685-689 (1997); published online EpubAug (
4. S. S. Shankar, R. R. Shankar, L. A. Mixson, D. L. Miller, H. O. Steinberg, C. R. Beals, D. E. Kelley, Insulin secretory effect of sitagliptin: assessment with a hyperglycemic clamp combined with a meal challenge. *American Journal of Physiology-Endocrinology and Metabolism* **314**, E406-E412 (2018)10.1152/ajpendo.00238.2017).
