# Supplementary material for: Berberine is an insulin secretagogue targeting the KCNH6 potassium channel
Source: Nat Commun. 2021 Sep 23;12:5616. doi: 10.1038/s41467-021-25952-2 (PMC8460738; doi:10.1038/s41467-021-25952-2)
Supplement: Supplementary file 3 — Reporting Summary [file 41467_2021_25952_MOESM3_ESM.pdf]

nature research

Corresponding author(s): Jin-Kui Yang

Last updated by author(s): Jul 19, 2021

Reporting Summary

Nature Research wishes to improve the reproducibility of the work that we publish. This form provides structure for consistency and transparency in reporting. For further information on Nature Research policies, see our Editorial Policies and the Editorial Policy Checklist.

Statistics

For all statistical analyses, confirm that the following items are present in the figure legend, table legend, main text, or Methods section.

n/a	Confirmed	
☐	☒	The exact sample size (n) for each experimental group/condition, given as a discrete number and unit of measurement
☐	☒	A statement on whether measurements were taken from distinct samples or whether the same sample was measured repeatedly
☐	☒	The statistical test(s) used AND whether they are one- or two-sided<br>*Only common tests should be described solely by name; describe more complex techniques in the Methods section.*
☐	☒	A description of all covariates tested
☐	☒	A description of any assumptions or corrections, such as tests of normality and adjustment for multiple comparisons
☐	☒	A full description of the statistical parameters including central tendency (e.g. means) or other basic estimates (e.g. regression coefficient) AND variation (e.g. standard deviation) or associated estimates of uncertainty (e.g. confidence intervals)
☐	☒	For null hypothesis testing, the test statistic (e.g. F, t, r) with confidence intervals, effect sizes, degrees of freedom and P value noted<br>*Give P values as exact values whenever suitable.*
☒	☐	For Bayesian analysis, information on the choice of priors and Markov chain Monte Carlo settings
☒	☐	For hierarchical and complex designs, identification of the appropriate level for tests and full reporting of outcomes
☒	☐	Estimates of effect sizes (e.g. Cohen's d, Pearson's r), indicating how they were calculated

Our web collection on statistics for biologists contains articles on many of the points above.

Software and code

Policy information about availability of computer code

Data collection	PatchMaster v2x80 software was used to collect data from patch-clamp experiments. GraphPad Prism 8.0 and Excel were used for data collection. LabChart 8.1.19 software was used to collect data from mice ECG experiments.
Data analysis	Graphpad prism 8.0 was used for statistical analyses. Image Lab 6.0 software was used for western-blotting analyses. Clampfit 10.3 software was used for patch-clamp data analyses. ImageJ 1.52a software was used for confocal microscopy images analyses. LabChart 8.1.19 software was used to analyze mice ECG data.

For manuscripts utilizing custom algorithms or software that are central to the research but not yet described in published literature, software must be made available to editors and reviewers. We strongly encourage code deposition in a community repository (e.g. GitHub). See the Nature Research guidelines for submitting code & software for further information.

Data

Policy information about availability of data

All manuscripts must include a data availability statement. This statement should provide the following information, where applicable:

- Accession codes, unique identifiers, or web links for publicly available datasets
- A list of figures that have associated raw data
- A description of any restrictions on data availability

All data underlying the clinical trial results are available in the “Supplementary Dataset: Source data” file linked to this article. The full scans of all the immunoblot images are available in the “Supplementary Dataset: Source data” file linked to this article. Other data that support the findings of this study are available from the corresponding author upon reasonable request.

April 2020

Field-specific reporting

Please select the one below that is the best fit for your research. If you are not sure, read the appropriate sections before making your selection.

- [x] Life sciences
- [ ] Behavioural & social sciences
- [ ] Ecological, evolutionary & environmental sciences

For a reference copy of the document with all sections, see nature.com/documents/nr-reporting-summary-flat.pdf

Life sciences study design

All studies must disclose on these points even when the disclosure is negative.

Sample size	No statistical method was used to calculate sample size. Sample size was determined based on preliminary experiments or previous publications (PMID: 9279533, 12196435, 29138226) and a sufficient number of samples needed to obtain definitive results. For in vivo experiments,at least 3 animals per group were used and experiments were performed at least three times to ensure reproducibility. For in vitro experiments, at least 3 biologically independent samples were used and repeated at least three times.
Data exclusions	No samples were excluded from analysis.
Replication	For in vivo experiments,at least 3 animals per group were used and experiments were performed at least three times to ensure reproducibility. For in vitro experiments, at least 3 biologically independent samples were used and repeated at least three times.
Randomization	Fasting glucose and body weight were measured before treatments. After, mice were allocated to groups ensuring same average body weight and glucose.
Blinding	Clinical trial was double-blinded. Data collection and analysis of other experiments were not performed blind.

Reporting for specific materials, systems and methods

We require information from authors about some types of materials, experimental systems and methods used in many studies. Here, indicate whether each material, system or method listed is relevant to your study. If you are not sure if a list item applies to your research, read the appropriate section before selecting a response.

Materials & experimental systems

n/a	Involved in the study	
☐	☒	Antibodies
☐	☒	Eukaryotic cell lines
☒	☐	Palaeontology and archaeology
☐	☒	Animals and other organisms
☐	☒	Human research participants
☐	☒	Clinical data
☒	☐	Dual use research of concern

Methods

n/a	Involved in the study	
☒	☐	ChIP-seq
☒	☐	Flow cytometry
☒	☐	MRI-based neuroimaging

Antibodies

Antibodies used	FLAG Sigma-Aldrich, St.Louis, MO, USA F3165 AB_259529, 1:2000 for western-blot.<br>β-actin Cell Signaling Technology, Danvers, MA, USA 58169 AB_42750839, 1:2000 for western-blot.<br>GAPDH Sigma-Aldrich, St.Louis, MO, USA G9545 AB_796208, 1:2000 for western-blot.<br>KCNH6 Sigma-Aldrich, St.Louis, MO, USA SAB2104242 SAB2104242, 1:100 for immunohistochemistry, 1:1000 for western-blot.<br>Insulin Abcam, Burlingame, CA, USA ab6995 AB_305690, 1:200 for immunohistochemistry.<br>Secondary antibody: Alexa Fluor 555 goat anti-rabbit IgG Invitrogen, South San Francisco, CA, USA A32732 AB_2633281, 1:500 for immunohistochemistry.<br>Secondary antibody: Alexa Fluor 488 goat anti-mouse IgG Invitrogen, South San Francisco, CA, USA A32723 AB_2633275, 1:500 for immunohistochemistry.<br>Na-K-ATPase, Cell Signaling Technology, MA, USA 3010S AB_2060983, 1:2000 for western-blot.
Validation	All antibodies were validated in our previous studies (PMID: 30590050, 32918525) and other publications.

Eukaryotic cell lines

Policy information about cell lines

Cell line source(s)	INS-1 cells,HEK293T and MIN6 cells were obtained from Cell Resource Center, Chinese Academy of Medical Sciences, Beijing, China.
Authentication	INS-1 and MIN6 have been authenticated by measuring insulin secretion.<br>293T cells have been authenticated by their ability to produce adenovirus.
Mycoplasma contamination	Mycoplasma contamination was not assayed.
Commonly misidentified lines (See ICLAC register)	No commonly misidentified cell lines were used.

Animals and other organisms

Policy information about studies involving animals; ARRIVE guidelines recommended for reporting animal research

Laboratory animals	C57BL/6J mice (strain code: 027) were purchased from Vital River Laboratories (Beijing, China). Both male and female mice were used at the indicated ages. βKO mice were sacrificed at the age of 6-8 weeks and littermates from the same breeding pair were used as controls. HFD-KO mice were provided with HFD beginning at 4 weeks of age for 8 weeks.
Wild animals	The study did not include wild animals.
Field-collected samples	The study did not include field-collected samples
Ethics oversight	Animal experiments followed the national ethical guidelines implemented by our institutional Animal Care and Use Committee and were approved by the Ethical Review Committee at the Institute of Zoology, Capital Medical University, China.

Note that full information on the approval of the study protocol must also be provided in the manuscript.

Human research participants

Policy information about studies involving human research participants

Population characteristics	Fifteen healthy male research subjects aged 18-45 yrs with a body mass index (BMI) of 18-25 kg/m2, normal oral glucose tolerance, normal blood pressure, normal laboratory values for HbA1c and kidney and liver functions, no family history of diabetes and no use of medications were enrolled in this study.
Recruitment	All volunteers were enrolled by the researchers.
Ethics oversight	The study was conducted with the approval of the Ethics Committee of Beijing Tongren Hospital, Capital Medical University (TRECKY2019-037).

Note that full information on the approval of the study protocol must also be provided in the manuscript.

Clinical data

Policy information about clinical studies

All manuscripts should comply with the ICMJEguidelines for publication of clinical research and a completedCONSORT checklist must be included with all submissions.

Clinical trial registration	ClinicalTrials.gov Registration No. NCT03972215
Study protocol	The full trial protocol is provided in Supplementary Note 2. This was a randomized, double-blind, placebo-controlled, two-period crossover study. The subjects were studied on two separated experimental days, at 14 days apart, and randomized to the order of BBR or placebo. The subjects were instructed to maintain their usual lifestyle and took no alcohol or cigarettes 3 days before the study. In each experimental day, following an overnight fast, subjects received a single oral dose of BBR 1g or matching placebo 1hr before the start of clamp study. Fasting blood glucose concentrations before and after drug treatment were measured to assess the effect of BBR on basal glucose level. Then they underwent a 160min hyperglycemic clamp at at a baseline blood glucose level +6.9 mmol/L as the target level. Two catheters were inserted into the antecubital veins of both arms for infusions and blood collection, and the arm was warmed to ~55℃ with a heating pad to obtain arterialized venous blood.<br>Blood samples for determinations of glucose, insulin and proinsulin C-peptide were obtained at intervals throughout the clamp study. Hyperglycemic clamp was initiated with a 14-min priming dose of 20% glucose (Baxter, Shanghai, China) to quickly increase blood glucose to the target level, and then the glucose infusion rate (GIR) was adjusted to maintain the glucose level. Plasma glucose was measured every 5 min throughout the study (EKF Biosen C-Line Glucose and Lactate analyzer, Cardiff, UK).<br>Glycated hemoglobin A1c (HbA1c) was measured by a high-performance liquid chromatography (HPLC) instrument, VARIANT II (Bio-rad, California, USA). Insulin and C-peptide were determined using IMMUNLITE-2000 (Siemens, Malvern, PA, USA). These biochemical measurements had been participated in the Chinese Ministry of Health Quality Assessment Program.
Data collection	The first volunteer was included on the 10/01/2019, the last experiment was completed on the 01/20/2020 and the study was finished on the 02/17/2020 after the follow-up period and statistical analysis. All experiments were performed at Beijing Tongren

Hospital, Capital Medical University, Beijing, China. The subjects were studied on two separated experimental days, at 14 days apart, and randomized to the order of BBR or placebo.In each experimental day, following an overnight fast, subjects received a single oral dose of BBR 1g or matching placebo 1hr before the start of clamp study. Fasting blood glucose concentrations before and after drug treatment were measured to assess the effect of BBR on basal glucose level. Then they underwent a 160min hyperglycemic clamp at at a baseline blood glucose level +6.9 mmol/L as the target level. Plasma glucose was measured every 5 min throughout the study

Outcomes

Primary Outcome Measure:
1. Differences of serum insulin levels between BBR and placebo treatment groups during the hyperglycemic clamp study. To compare the mean serum insulin levels in the two groups during hyperglycemic clamp study.
2. Differences of serum C-peptide levels between BBR and placebo treatment groups during the hyperglycemic clamp study. To compare the mean serum C-peptide levels in the two groups during hyperglycemic clamp study.
Secondary Outcome Measure:
3. Differences of glucose infusion rates between BBR and placebo treatment groups during the hyperglycemic clamp study. To compare the mean glucose infusion rates in the two groups during hyperglycemic clamp study.
4. Differences of blood glucose levels between BBR and placebo treatment groups during the hyperglycemic clamp study. To compare the mean blood glucose levels in the two groups during hyperglycemic clamp study.
Other pre-specified Outcome Measures:
5. Heart rate and QT-interval duration using electrocardiogram after drug treatment.
